# Effect of Oleylamine on the Surface Chemistry, Morphology, Electronic Structure, and Magnetic Properties of Cobalt Ferrite Nanoparticles

**DOI:** 10.3390/nano12173015

**Published:** 2022-08-31

**Authors:** Sumayya M. Ansari, Bhavesh B. Sinha, Debasis Sen, Pulya U. Sastry, Yesh D. Kolekar, C. V. Ramana

**Affiliations:** 1 Department of Physics, Savitribai Phule Pune University, Pune 411 007, Maharashtra, India; 2 National Center for Nanoscience and Nanotechnology, University of Mumbai, Mumbai 400 032, Maharashtra, India; 3 Bhabha Atomic Research Centre (BARC), Solid State Physics Division, Mumbai 400 085, Maharashtra, India; 4 Homi Bhabha National Institute, Anushaktinagar, Mumbai 400 094, Maharashtra, India; 5 Centre for Advanced Materials Research (CMR), University of Texas, El Paso, TX 79968, USA

**Keywords:** CoFe_2_O_4_, oleylamine, solvothermal, crystal structure, magnetic properties

## Abstract

The influence of oleylamine (OLA) concentration on the crystallography, morphology, surface chemistry, chemical bonding, and magnetic properties of solvothermal synthesized CoFe_2_O_4_ (CFO) nanoparticles (NPs) has been thoroughly investigated. Varying OLA concentration (0.01–0.1 M) resulted in the formation of cubic spinel-structured CoFe_2_O_4_ NPs in the size-range of 20–14 (±1) nm. The Fourier transform spectroscopic analyses performed confirmed the OLA binding to the CFO NPs. The thermogravimetric measurements revealed monolayer and multilayer coating of OLA on CFO NPs, which were further supported by the small-angle X-ray scattering measurements. The magnetic measurements indicated that the maximum saturation (M_S_) and remanent (M_r_) magnetization decreased with increasing OLA concentration. The ratio of maximum dipolar field (H*_dip_*), coercivity (H_C_), and exchanged bias field (H_ex_) (at 10 K) to the average crystallite size (D_xrd_), i.e., (H*_dip_/*D_xrd_), (H_C_*/*D_xrd_), and (H_ex_*/*D_xrd_), increased linearly with OLA concentration, indicating that OLA concurrently controls the particle size and interparticle interaction among the CFO NPs. The results and analyses demonstrate that the OLA-mediated synthesis allowed for modification of the structural and magnetic properties of CFO NPs, which could readily find potential application in electronics and biomedicine.

## 1. Introduction

Magnetic material, chemical, and biological architectures at the nanoscale dimensions have introduced revolutionary healthcare and medical therapy trends [1]. Because of its potential biomedical applications such as in bioimaging [2], cell labeling [3], magnetic hyperthermia [4], and drug delivery [5], magnetic nanoparticles (MNP) are one of the most appealing materials. ‘Size control’ in biomedical applications permits nanoparticles to circulate through the bloodstream, infiltrate through cell membranes, and bypass immune system detection [1,6]. Furthermore, the particle surface must be adequately tailored for biomedical and healthcare applications to achieve better colloidal stability in physiological fluids, drug encapsulation ability, and specific targeting to ensure proper interaction with cells or tissues [7]. Due to the diversity in the chemistry and physics of various biomolecules, designing a suitable surface modification method is challenging [8], especially one that balances the intermolecular force between the biomolecules and the outer layer of MNP [9,10]. Thus, the preparation of monodisperse, size-controlled MNPs is fundamentally important and inspiring, as their properties are strongly dependent on dimensions. [9,10]. Cobalt ferrite (CoFe_2_O_4_ (CFO)) nanoparticles (NPs) have been extensively explored [11,12] due to their potential applications in a variety of fields. Some of these include luminescence, molecular imaging, hyperthermia [13,14], electromagnetic wave absorption performance [15], theranostic applications [16], drug delivery [17] magnetic resonance imagining [18], catalysis [19], and field emission-based device applications [20]. However, controlling the crystal size, morphology, and composition of CFO nanomaterials is the key to achieving these applications [19]. For instance, as demonstrated by Dippong et al. [19], the density and porosity of the CFO nanomaterials are affected based on the synthetic approach and ionic size difference of the dopants considered [19]. In addition, tuning the size–shape correlation while maintaining thermal and chemical stability is essential for practical applications. This is generally accomplished by surface modification [21]. Magnetic nanoparticles tend to agglomerate due to Van der Waals forces and magnetic dipolar interactions, which can be decreased by encapsulating the magnetic nanoparticles with suitable capping agents [22,23]. Moreover, the size, shape, dispersibility in a biological medium, and chemical stability of surface-functionalized MNPs are the critical parameters for determining their applicability in biomedicine [24,25]. In this context, coating with oleic acid [26,27,28] and oleylamine (OLA) [29] is interesting for magnetic CoFe_2_O_4_ NPs. Oleylamine, alkyl amine with a low affinity for transition metals, can operate as a solvent, surfactant, and reducing agent all in one, minimizing the need for additional reagents. Also, as reported by Georgiadou et al., OLA is an inexpensive, easily handled surfactant due to its liquid state, while its high boiling point and reducing ability offer stability under the harsh solvothermal conditions with no need of an extra solvent [9]. Additionally, the multifunctional role of OLA as a versatile and flexible reagent in synthesis of nanoparticle appears [9] to have a potential for utilization in the large-scale production of magnetic nanoparticles for a wide variety of application. However, to date, only a few research efforts have reported successfully generating CFO NPs with oleylamine [9,29,30,31]. Furthermore, to our knowledge, fundamental scientific knowledge on the effect of OLA concentration on the attributes of CFO NPs, such as particle size, shape, and magnetic properties, is rare. In this context, CFO nanoparticles were synthesized via a one-step, ecofriendly, cost-effective solvothermal route at lower temperatures (180 °C) by simply varying the OLA concentration. As widely known to the scientific community, the solvothermal method is environmentally friendly and is easier to conduct, as it does not need toxic chemicals and oxygen-free procedures. Compared to other methods, the solvothermal synthesis route is a simple one with a high rate of reaction with a low temperature of operation, resulting in uniform size distribution. Therefore, we adopted this method for the synthesis of the CFO NPs with varying OLA concentrations so as to produce CFO NPs with variable size and magnetic properties. We explored the effect of OLA concentration on the physical and chemical properties of CFO NPs to bridge the information gap and provide a deeper scientific understanding. As presented and discussed elaborately, the present paper explores the interplay between structural characteristics, electronic structure, interparticle interactions, and magnetic properties.

## 2. Materials and Methods

The CoFe_2_O_4_ NPs were synthesized using a modified solvothermal [32] route by varying the OLA concentration. Details of synthesis are reported in elsewhere [27]. Briefly, the ethylene glycol solution was used as a solvent, and 0.01 M OLA solution was added and ultrasonicated for one hour. After that, stoichiometry molar (1:2) amounts of cobalt (II) nitrate and iron (III) nitrate were added to the ethylene glycol solution and were stirred well for 1 hr. The chemical solution was subjected to solvothermal treatment at 180 °C for 24 h. CoFe_2_O_4_ NPs with 0.01, 0.05, and 0.1 M OLA were synthesized using the same approach and named CoFe1, CoFe2, and CoFe3, respectively. For clarity purposes, the number of moles of all reagents used in the synthesis are summarized in Table 1.

The morphology of the pristine samples was explored using field-emission scanning electron microscopy (Fe-SEM, Karl ZEISS JEOL, Akishima, Tokyo, JAPAN). The size distribution of NPs was calculated using Image-J software. The compositional study of pristine NPs was carried out using energy-dispersive X-ray spectrometry (EDS). Small-angle X-ray-scattering (SAXS) measurements were carried out using a Rigaku small-angle goniometer mounted on a rotating anode X-ray generator. Details are mentioned in supporting information. Fourier transform infrared spectroscopy (FTIR) studies were performed on pure OLA, as well as CFO NPs produced with a varied OLA content to better understand the oleylamine adsorption mechanism on the surface of CFO NPs. The FTIR spectra of powder samples were acquired in the range of 400–4000 cm^−1^ using the JASCO-6100 spectrometer. The structural analyses of pristine NPs were carried out using an X-ray powder diffractometer (D8-Advanced Bruker,) with Cu K_α_ radiation (λ ~ 1.5406 Å) at a scanning rate of 4° min^−1^, over a 2θ range of 20° to 80°. The Rietveld refinement method was used to accomplish detailed structural characterization, utilizing ICSD data with the collection code: 109044. The thermogravimetric analysis was carried out with the help of the (TGA, METTLER TOLEDO. The X-ray photoelectron spectroscopy (XPS) measurements were performed by Al-K_a_ (1486.6 eV) X-ray source. The Evercool II PPMS-6000 from Quantum Design was used to conduct the magnetic measurements. Magnetic fields up to 90 kOe were used to measure the magnetization hysteresis (M-H) loops at 300 K and 5 K. Under a 100 Oe applied magnetic field and a temperature range of 10 to 398 K, the temperature dependence of magnetization, i.e., M (T), was measured.

## 3. Results and Discussion

### 3.1. Chemical Composition, Morphology, and Inter-Particle Structure

**Figure 1:**
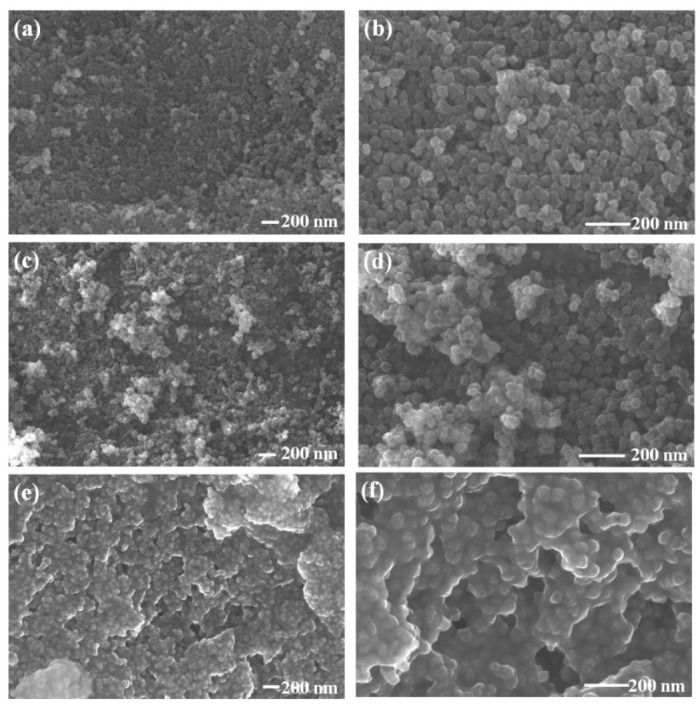
SEM images of CoFe1 (**a**,**b**), CoFe2 (**c**,**d**), and CoFe3 (**e**,**f**) samples.

**Figure 2:**
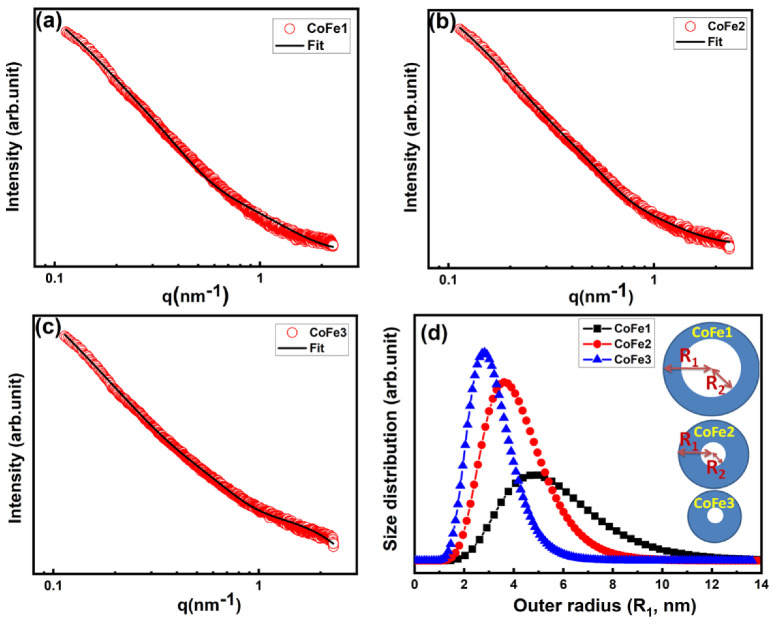
SAXS profile for CoFe1 (**a**), CoFe2 (**b**), CoFe3 (**c**) and corresponding size distributions of CFO NPs (**d**).

The EDS analysis helped to identify various elements present in the sample in addition to information on the chemical homogeneity [33,34]. In order to probe accurately the chemical information and to understand the effect of OLA, the EDS spectra of the CFO NPs were considered. The EDS spectra (Figure S1; Supporting Information) for OLA functionalized CFO NPs indicated Co: Fe’s desired stoichiometry (1:2). The presence of C and N was expected due to the capping of OLA on the surface of NPs, which also confirmed OLA presence in all the samples. The effect of OLA concentration on the morphology of pristine CFO NPs is evident in FESEM images presented in Figure 1a–f. Evidently, at lower concentration, a spherical shape of CFO NPs that are well-separated from each other are obtained. However, CFO NPs prepared with higher OLA concentration (0.1 M) exhibited a very compact arrangement of CFO NPs that created layer appearance. The uniform size distribution was evident for all the samples. The median diameter obtained is ~39.21 nm and 36.14 nm for CoFe1, and CoFe2 samples, respectively. The size reduction was noted as a function of OLA concentration. However, it was difficult to quantify and predict the size using SEM micrograph for CoFe3 due to the compact arrangement of NPs. Therefore, further analysis for size of NPs was performed using SAXS. As shown in Figure 2a–c, the scattering profiles were very similar for all the samples. The mesoscopic density fluctuations in a material are represented by scattered intensity *I* (*q*), where q is the scattering vector [35].

In the present case, the scattering occurs due to density fluctuations arising from the primary spherical CFO NPs. The scattering profiles of all the samples have been analyzed based on the polydisperse spherical shell particle model under monodispersed approximation [36,37]. Figure 2a–c shows the scattered intensity profiles and the fitted curves for all the samples. The fractal dimension of the three samples is ~3, which agrees with the standard value for spherical shape. The structural parameters obtained from the SAXS analysis are listed in Table S1. Two key points emerged from the SAXS study of OLA-coated samples. (i) There were two contributions in all the samples: (a) naked spherical shell nanoparticles with a mass fractal structure factor and (b) inhomogeneities on a length scale of 2 nm, which could be attributed to OLA capping over the NPs. (ii) As the concentration of OLA rose, the average size of CFO NPs and monomer radius (r_o_) decreased. The size distribution patterns obtained from SAXS analysis are shown in Figure 2d, which were consistent with the XRD results. The outer radius of NPs for CoFe1, CoFe2, and CoFe3 samples was 5.48 nm, 4.00 nm, and 3.05 nm, respectively. For CoFe1, the difference between outer and inner radius, i.e., ΔR, was found to be 2 nm, indicating that OLA is monolayer coated (OLA length size 1.97 nm). The presence of ΔR > 2 nm in CoFe2, and CoFe3, indicated that OLA is multilayered. These findings are consistent with the TGA studies reported in the following section.

### 3.2. FTIR Analysis 

**Figure 3:**
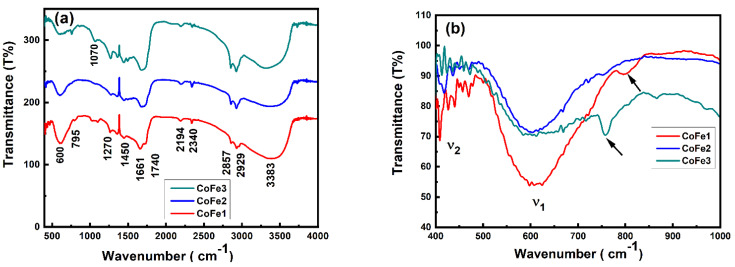
FTIR spectra of OLA coated CFO NPs (**a**) for 400–4000 cm^−1^ and (**b**) magnified data from 400–1000 cm^−1^ for clarification.

To further assess the chemical bonding and structural quality of CFO NPs, we relied on spectroscopic characterization, particularly FTIR measurements. Note that the spectroscopic characterization using FTIR and/or Raman scattering provides direct information on the chemical bonding and dopants (if any) [38,39]. In the present case, the FTIR measurements allowed us to confirm the OLA binding to the CFO NPs. For reference purposes, the FTIR data of pure OLA is shown in Figure S2. Peak identification was carried out by the literature [40]. The vibrational modes observed for pure OLA are summarized in Table S2. Figure 3a presents the FTIR spectra of CFO NPs prepared with different concentration of OLA. The −CH_2_− scissoring and NH_2_ scissoring peaks at 1450 cm^−1^ and 1661 cm^−1^, respectively, were visible. Their appearance indicated the presence of OLA molecules on the surface of CFO NPs. The observed peaks matched with the literature. Moreover, the bending vibration of C–N was observed at 1070 cm^−1^ only for CoF1 and CoFe3. All the samples exhibited -CH_2_ symmetric and asymmetric stretching vibrations at 2857 and 2929 cm^−1^, respectively, revealing the absorption of the oleyl group onto the surface [41,42]. The characteristic peaks of OLA at 1593 and 3300 cm^−1^ were not detected. This indicates that no free OLA existed at the surface [43]. However, for all the samples, a broad peak located at 3423–3383 cm^−1^ was not expected. It was assigned to the ν(N–H) stretching of the NH_2_ group [44]. Furthermore, as shown in Figure 3b two signature absorption bands for ferrite NPs were observed. The first absorption band (ν_1_) observed at 612–600 cm^−1^ was assigned to the stretching vibrations of tetrahedral metal (Fe^3+^)–oxygen bond. The second band (ν_2_) positioned at 407–420 cm^−1^ was caused by the octahedral metal-oxygen bond vibrations at octahedral sites. In addition, slight splitting of the octahedral absorption band near ν_2_ was observed as it was beyond the detection limit of our FTIR equipment (<400 cm^−1^).

### 3.3. Crystal Structure 

**Figure 4:**
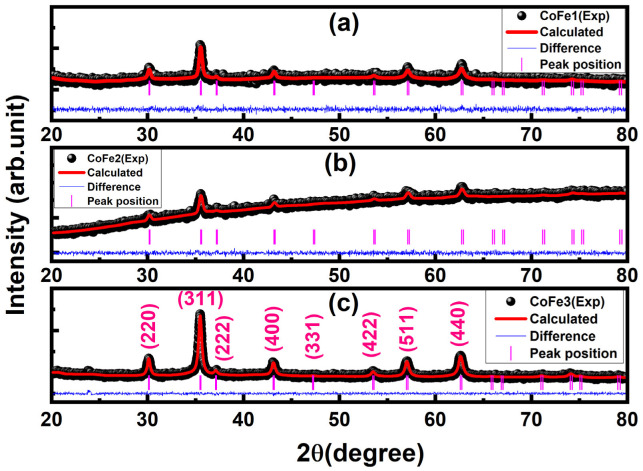
Rietveld refined XRD patterns of CoFe1 (**a**), CoFe2 (**b**) and CoFe3 (**c**) samples.

Figure 4 shows the X-ray diffraction pattern for all samples, along with Rietveld refinement. Tables S3 and S4 list the structural parameters gained after refinement. All the samples were crystallized in the cubic spinel structure (JCPDS file No. 221086), according to the refinement data. The lattice constant of functionalized CFO NPs (8.391 Å) was lower than that of bulk CFO. Size factors, such as surface dipole interactions, surface tension, and cation charge distribution inside the nano-crystallite, were attributed to the lower lattice constant [10]. With increasing OLA concentration, the diffraction peaks became broader, reflecting a reduction in crystallite size. As OLA content increased, the average crystallite size dropped from 20 nm to 14 nm (±1 nm).

**Figure 5:**
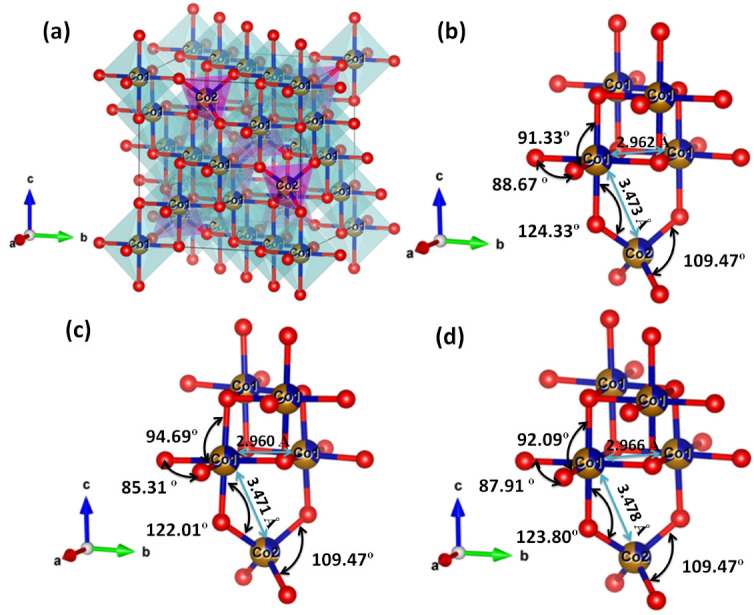
The crystal structure of the CoFe1 sample is shown (**a**), where octahedral, tetrahedral, and oxygen atoms are highlighted in blue, violet, and red color, respectively. The bond angles and neighborhoods around the tetrahedral and the octahedral sites in CoFe1 (**b**), CoFe2 (**c**), and CoFe3 samples (**d**) are illustrated.

Using the Rietveld refinement parameters, the unit cell model for all samples was constructed using the VESTA software. The results for CoFe1 are shown in Figure 5a. All the samples were found to be made up of a cubic close-packed array of oxygen anions occupying the 32e position at (0.247, 0.247, 0.247). The cations (Fe^+3^, Co^+2^; Fe1 and Co1) were octahedral 16c site {B-sites} with trigonal 3 m point symmetry. The cations (Fe^+3^, Co^+2^; designated as Fe2 and Co2) occupied cubic 43 m point symmetry tetrahedral 8b sites [A-sites].

It is noticeable that (*i*) the refinement of diffraction data for CoFe1, CoFe2, and CoFe3 exhibited deviation from the ideal inverse cation distribution i.e., [Fe^+3^]{Co^+2^Fe^+3^}O^−2^ by transferring some Co^+2^ cations from octahedral (B-site) to tetrahedral (A-site) i.e., [Co^+2^_(x)_ Fe^+3^]_Tet_{Co^+2^_(1−x)_Fe^+3^}_Oct_O^−2^. (*ii*) Importantly, the occupancy and Wyckoff position of metal cations were nearly identical for all samples. However, different occupancy was observed for oxygen anion for all the samples. Therefore, it is reasonable to postulate that OLA might have formed the coordination with lattice oxygen, which can disturb the overall charge compensation of CFO lattice. It may have generated some lattice defects that led to the observed variation in the lattice parameters. Furthermore, it can be observed that the bond length observed at A and B sites and inter-cation distances slightly changed as OLA concentration changed (see, Table 2). Accordingly, slight local distortion was observed in Co1–O–Co2 bond angle (for all the samples) compared to the standard value of 125.26° reported for ideal spinel structure. Furthermore, the O–Co1–O bond angle deviated to 88.67°, 85.31°, and 87.91° as opposed to the O–Fe1–O bond angle of 91.33°, 94.69°, and 92.09° observed for CoFe1, CoFe2, and CoFe3 samples, respectively. The O–Co1–O and O–Fe1–O bond angle values deviated from an ideal spinel value of 90°. This distortion can be attributed to the decrease in crystallite size. Notably, the observed O–Co2–O bond angle matched the ideal value of 109.47° for all the samples. Thus, it is evident from the observed values of bond angle and bond length that all the OLA functionalized CFO NPs were stabilized in the cubic structure with slight local distortion at B sites. Therefore, the (*i*) absence of local distortion at tetra- and octahedral sites and (*ii*) decrease in particle size as a function of OLA concentration strongly supported the significant role of oxygen occupancy and capping of OLA with CFO NPs. As expected, and postulated, OLA controlled the nucleation and growth of NPs. Consequently, we can expect the modification of magnetic properties of CFO NPs as a function of OLA concentration.

### 3.4. Electronic Structure and Surface Chemistry

**Figure 6:**
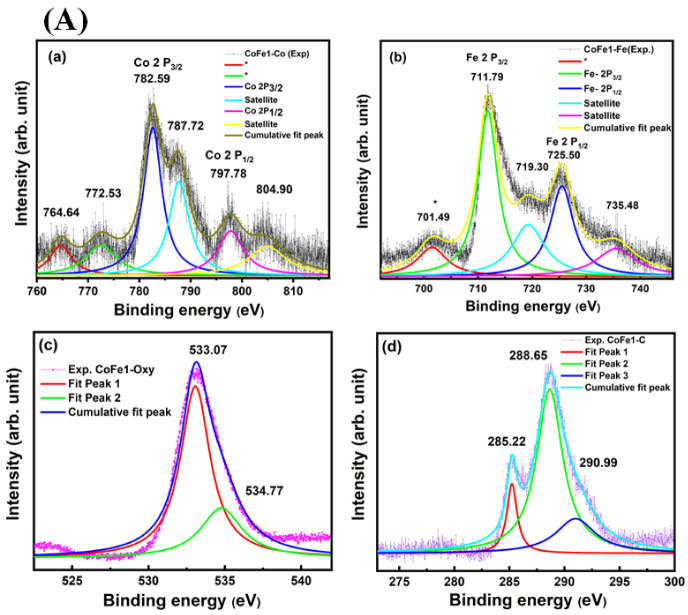

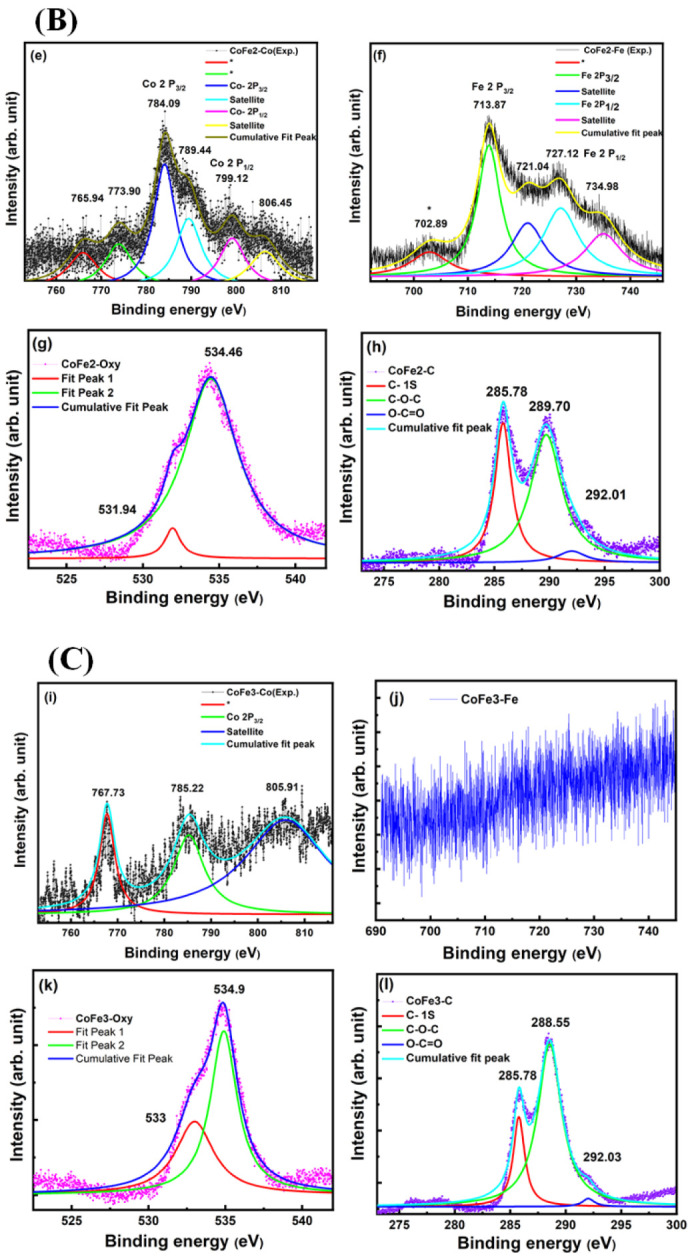
XPS spectra of CoFe1 (**A**), CoFe2 (**B**) and CoFe3 (**C**) showing the binding energy and chemical states of Co 2p, Fe 2p, O 1s, and C for CoFe1 (**a**–**d**), CoFe2 (**e**–**h**) and CoFe3 (**i**–**l**) samples.

Finally, to probe the chemistry of the CFO NPs samples and the effect of OLA on the electronic structure, the samples were analyzed using XPS. Specifically, an attempt was made to establish the oxidation states of Co and Fe in the CoFe_2_O_4_ NPs using core-level spectra of the respective elements. The XPS data are shown in Figure 6. The survey XPS spectra of all the samples are presented in Figure S3. The XPS data indicated the presence of respective O 1s, Fe 2p, Co 2p, N 1s, and C 1s peaks. For the CoFe1 sample, the XPS spectrum of Fe 2p is presented in Figure 6A. The binding energy (BE) position and separation of Fe 2 p3/2 (711.79 eV) and 2 p1/2 (725.49 eV) peaks, and the presence of corresponding shakeup satellites at 719.30 and 735.48 eV, respectively, characterized Fe ions in their highest oxidation state (+3) [45,46]. Furthermore, it has been reported that the BE Fe^+2^ is at 709.9 eV, which was not detected in all the samples confirming the absence of Fe^2+^ state [47]. The unassigned shoulder at BE~701.49 and 702.89 eV, respectively, for CoFe1 and CoFe2, may be due to the metallic state of Fe as the BE for metallic Fe is expected ~707 eV [48,49,50].

The XPS core level data of Co 2p also evidenced that the Co ions exist in a +2 chemical state. The peaks at 782.59 eV and 804.90 eV with its satellite at 787.72 eV and 797.78 eV, respectively, indicated Co in +2 state [51,52]. Moreover, the unassigned shoulder at around (764.64 eV, 772.53 eV), (765.94 eV and 773.90 eV) and 767.73 eV observed for CoFe1, CoFe2 and CoFe3, respectively [48,49,50], may have been due to the metallic state of Co. Furthermore, the O 1s XPS spectrum shown in Figure 6 (Ac, Bg, Ck) was divided into two peaks. CoFe1 sample showed the peaks were positioned at binding energies of 533.07 eV and 534.77 eV. The most intense peak at 533.07 eV corresponded to O2− in the CFO spinel crystal lattice [52]. However, in CFO NPs, BE~534.77 eV was ascribed as the surface and near-surface defect sites with low oxygen coordination, often formally described as O− species [53]. With an increase in OLA concentration, the following were noted: (i) The most intense peak shift was from 533.07 eV (CoFe1) to 534.46 eV, (CoFe2) and 534.9 eV (CoFe3). (ii) The peak became asymmetric at lower BE for CoFe2 and CoFe3 samples compared to CoFe1 sample. This observation suggests that they were caused mainly by the chemisorbed oxygen species because the smaller sized nanoparticles generally exhibited a higher adsorption capability [54]. Furthermore, we could verify the presence of OLA capping on the surface of CFO NPs in the presence of carbon and nitrogen peaks. Normally carbon peak originated at ~285 eV. It must be emphasized that the carbon peak in the XPS spectra may also have been due to adventitious carbon from the exposure of samples to air following the synthesis before being placed in the XPS system. Therefore, C 1s peak in XPS alone may have not provided direct confirmation of OLA. On the other hand, the presence of OLA capping on the surface of CFO NPs validated more directly FTIR results, which corroborated with the XPS to some extent. For all the three capped NPs, it resulted in an amine co-adsorbate that could be detected in the XPS N 1s region as a single peak found at 399.7 eV (Figure 6). The BE was comparable to literature values for amine-capped nanoparticles [55,56]. For the OLA-capped CFO NPs, the C 1s XPS region, shown in Figure 6 (Ad, Bh, Cl) indicated three different carbon-containing species. It has been reported that the C 1 s feature was composed of multiple peaks at lower binding energy, i.e., ~285.22 eV (for CoFe1) used for calibration. In the present case, the peak found at 285.22 eV, 285.73 eV, 285.78 eV for CoFe1, CoFe2, and CoFe3 was attributed to the carbon of the alkyl chains of oleylamine, which also agreed with the literature [57]. The peak observed at 288.65 eV, 289.70 eV, and 288.55 eV for CoFe1, COFe2, and CoFe3, respectively, was a combination of the C-N bond [58,59], C=O (~287.38 eV), and O–C=O (~288.88 eV) of oleylamine. The third peak observed at 290.99 eV, 292.01 eV and 292.03 eV for CoFe1, CoFe2 and CoFe3, respectively, corresponded to the Plasmon π-π* [57,58]. Remarkably, with an increase in OLA concentration, the peak position value of Co^+2^, Fe^+3^, O, and C was observed to shift towards the higher BE from CoFe1 to CoFe3, respectively. Poor signal originates for Fe^+3^ and Co^+2^ in CoFe3 compared to CoFe1 due to the fact that it was prepared with a higher concentration of OLA and exhibited the multilayer coating. Thus, FTIR, XPS, EDS, and TGA results validated and confirmed the OLA functionalization of the CFO NPs.

### 3.5. Thermal Behavior-Thermogravimetric (TGA) Analysis

**Figure 7:**
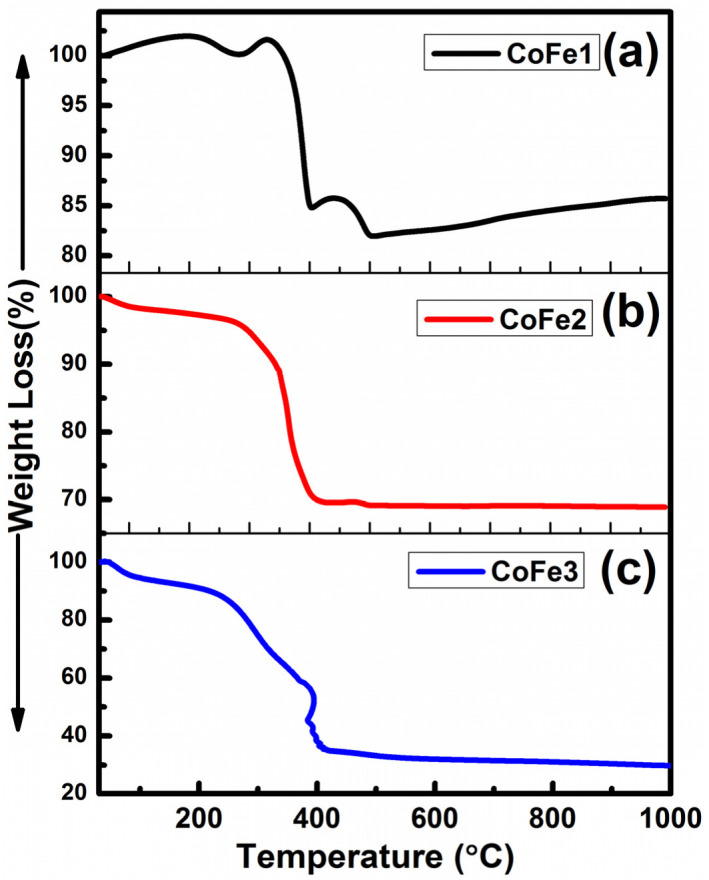
(**a**–**c**) Thermogravimetric curves obtained (under air atmosphere) for CFO NPs synthesized with variable OLA concentration.

The TGA data of the CFO NPs are presented in Figure 7. Moisture and volatile component loss were responsible for the initial weight loss observed in all samples in the 50–100 °C range. Because the breakdown temperature of OLA is 350 °C, the second weight loss occurred in the range of 100–400 °C due to breaking functional groups from the surfactant layer. CO and CO2 effluents from the sample accounted for the third weight loss, which occurred in the 400–600 °C range. The total weight loss noted for CoFe1, CoFe2 and CoFe3 was 18.12%, 31.06% and 68.74%, respectively. The increased percentage of weight loss with OLA concentration indicated the OLA effect on the CFO NPs’ surface. Note that the sample’s thermal behavior depends on structure, homogeneity, and composition. Decomposition products are released more rapidly when particles size is reduced. As the particle size was reduced, the surface area increased, allowing more water molecules to escape during heating [27]. When weight loss was less than 20%, the monolayer coating of surfactant was present, and when weight loss was greater than 20%, the multilayer coating of surfactant was present [27]. Thus, for CoFe1, it is reasonable to believe that the OLA forms a monolayer on individual NPs, but for CoFe2 and CoFe3, it is possible to suppose that the OLA forms a multilayer on individual NPs. OLA ligands per particle are ~369, 476, and 732 for CoFe1, CoFe2, and CoFe3. While the present scope of the work is more directed towards the CFO NPs synthesis and optimizing conditions towards realizing superior magnetic properties, Stefanescu et al. demonstrated an approach to realize CFO NPs in SiO_2_ matrix by thermal decomposition of carboxylate type precursors [60,61]. By heating the solutions metal nitrates-ethylene glycol, a redox reaction produced carboxylate anions, which reacted with Co(II) and Fe(III) cations to form coordinative compounds, which eventually resulted in CFO NPs. The average diameter varied 10–20 nm, depending on annealing temperature [60].

### 3.6. Magnetic Properties

**Figure 8:**
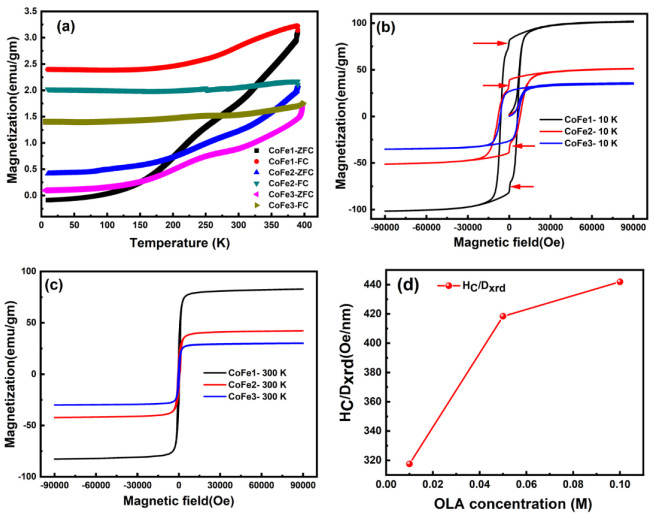
Zero field cooling (ZFC) and field cooling (FC) curves measured at 100 Oe (**a**), the M–H loops at 10 K (**b**), the M–H curves at 300 K (**c**), and HC/Dxrd versus OLA concentration (**d**) (at 10 K) of functionalized CFO NPs.

The ZFC-FC data (Figure 8a) shows a branching between the values of M_FC_ and M_ZFC_ that increased with decreasing temperature for all samples in the temperature range of 10–398 K at 100 Oe magnetic field. The irreversibility of the ZFC and FC curves begins far above 398 K, implying that all samples must have been above 398 K to overcome the superparamagnetic limit. This behavior indicated that the NPs have a highly anisotropic behavior. Only the CoFe1 sample had a negative ZFC magnetization (M_ZFC_) value at low temperatures (from 10 K to 86.15 K). This behavior can be attributed to a variety of factors, including structural phase transition changes in the sign of f-d exchange interaction spin reorientation and negative interaction coupling [62,63,64]. Furthermore, as the temperature rose, the M_ZFC_ value remained constant until it reached 25–50 K, at which point it began to approach the FC value. These findings were qualitatively consistent with the ferrimagnetic compositions based on CFO [60]. All samples showed a significant change in slope for the M_ZFC_ curve towards 200–300 K. CFO NPs produced with varying oleic acid concentrations exhibited similar activity [27]. The charge ordering, metal-insulator transition was responsible for this transformation. The negative result of M_ZFC_ in this study could be attributed to residual magnetization in the magnetometer that was not compensated by the 100 Oe field. The increase in volume anisotropy and interparticle interaction caused by molecular coating can cause the Tmax of M_ZFC_ to shift to higher temperatures [25]. The M_FC_ showed temperature-independent behavior after initially falling monotonically with decreasing temperature from 398 K to 100 K, corresponding to non-interacting areas. The temperature-independent behavior was related to dipolar contacts and interparticle coupling interactions, which resulted in finite-size interaction effects [25]. The M_FC_ fall more quickly from 397 K to 300 K (as reported for CoFe1), but more slowly from 300 K to 100 K. Furthermore, due to OLA diamagnetic susceptibility, the OLA-capped magnetic grains were restricted in ZFC conditions at lower temperatures and did not respond to the applied magnetic field [65,66].

Magnetization (M-H) loops measured at 10 K, and 300 K are shown in Figure 8b,c and magnetic parameters are presented in Table 3. Maximum saturation magnetization (M_S_) and remanent magnetization (M_r_) values (at 10 K and 300 K) were obtained for the CFO NPs prepared with 0.01 M of OLA. These M_S_ and M_r_ values were significant and higher compared to an ideal inverse CFO structure (80 emu/gm) and those reported for CFO NPs [2]. In order to understand the importance of these CFO NPs prepared by OLA and their magnetic behavior, a comparison of the magnetic parameters with those reported in the literature is presented in Table 4. Moreover, these values decreased for CFO NPs synthesized with higher OLA concentrations (0.05–0.10 M). The non-stoichiometric cation distribution among the octahedral and tetrahedral sites, as compared to the ideal spinel structure anticipated by XRD refinement, may account for the increase in M_S_ value for CoFe1. The inversion parameter obtained was 0.56. The parameter was obtained using the formula (Co_1−δ_Fe_δ_)[Co_δ_Fe_2−δ_]O_4_ to explain the cation distribution in the spinel structure of the CoFe_2_O_4_ NPs, in addition to assuming that Fe^+3^ and Co^+2^ ions have magnetic moments of 5 µ_B_ and 3 µ_B_, respectively. The partial inverse spinel crystal structure of CoFe_2_O_4_ NPs was indicated by this value of the inversion parameter. Similarly, improved magnetic property was reported for CFO NPs prepared with lower concentration (1–3 wt%) of polystyrene synthesized by solvothermal process [65,66]. The M_S_ and M_r_ values decreased with increasing OLA concentration. The M_S_ value reduction can be attributed mainly to the presence of OLA molecules and the smaller magnetic cores, surface disorder/spin canting at the NP surface [27,67]. The particle size is one of the critical facets in tuning the magnetic behavior of nanomaterials. The increased particle size in CoFe1 compared to CoFe2 and CoFe3 is evident from XRD and SAXS analyses. Thus, at lower (0.01 M) OLA concentrations, the larger size of NP lowers surface spin disorder, resulting in a considerable improvement in the M_S_ value.

**Figure 9:**
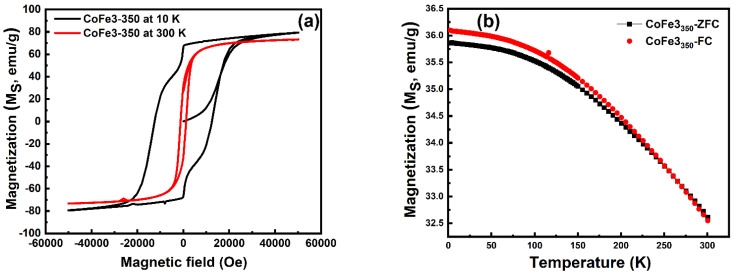
(**a**) the M–H loops at 10 K and 300 K; and (**b**) zero-field cooling (ZFC) and field cooling (FC) curves measured at 100 Oe for CoFe3-_350_ NPs.

Remarkably, jumps in the M-H curve at 10 K (Figure 8b) were noted only for CoFe1 and CoFe2 samples. This can be attributed to the low-temperature spin reorientation of surface spin and the crystal alignment of CFO nanospheres. Importantly, this jump was absent in the CFO NPs prepared with a higher (0.1 M) concentration of OLA (CoFe3). To get more insight, the CoFe3 sample was thermally treated at 350 °C to breakdown the OLA from CoFe3 NPs, and the resulting values are shown in Table 3. The M-H loops for the CoFe3 sample after thermal treatment (CoFe3_350_) at 10 K and 300 K are shown in Figure 9a. The M-H curve jumped at 10 K, which is interesting. As a result, we believe the observed kink was due to OLA coating efficiently reorienting the surface spin and interparticle interaction. Significantly, the M-H curve of the CoFe3_350_ sample revealed an increase in magnetic characteristics such as M_S_, M_r_, H_C,_ M_r_/M_S_, and K_E_ values (see Table 4). Furthermore, Figure 9b sheds the ZFC-FC curve for the CoFe3_350_ sample and notes that (*i*) the ZFC-FC curve of the CoFe3_350_ sample showed the absence of temperature-independent behavior, (*ii*) the bifurcation between the values of M_FC_ and M_ZFC_ was drastically decreased after the thermal treatment. An overall magnetic study implies that the interparticle interaction among the NPs is well controlled with OLA concentration.

At 10 K, the squareness ratio (R = M_r_/M_S_) is between 0.72–0.79. A trend toward cubic anisotropy is shown by high R values [27]. Moreover, all of the OLA-functionalized CFO NPs possessed coercivity (H_C_) of 6.09–8.00 kOe at 10 K, significantly higher than bulk CFO (~5 kOe at 5 K) [27]. Studies on the change in H_C_ with particle size have also been widely published [68,69,70,71,72,73,74]. Some researchers have proposed that H_C_ variations with particle size are due to a transition from single-domain to multi-domain behaviour. By altering the annealing temperature of CFO NPs, some investigations have found a non-monotonous variation in H_C_ with particle size [75,76]. When CFO NPS was prepared with 1, 2, 3, 4, and 5 wt% of polystyrene using the co-precipitation method, Vadivel et al. [63] found that H_C_ changes with particle size were not monotone. Therefore, the H_C_/D_XRD_ as a function of OLA content was also examined and is depicted in Figure 7d. The CoFe3 sample presented the highest H_C_/D_XRD_ values at 10 K, evidently due to the decreased particle size of NPs due to OLA concentration. This was further strongly supported by the fact that the after-heat treatment CoFe3_350_ sample showed the enhanced value of coercivity. Thus, the two points mentioned above imply that one can tune the coercivity of MNPs using the present approach. 

Furthermore, H_C_ is provided by Hc = 0.64 K_E_/M_S_ for randomly oriented, non-interacting spherical particles with cubic anisotropy, where K_E_ is the effective anisotropy constant. The K_E_ values were determined and are summarized in Table 3 using this relationship. At 10 K, the K_E_ values rose with particle size, as seen with Fe_3_O_4_ NPs [74], implying that the surface component of anisotropy (K_S_) played a modest role in these systems. For an increase in K_S_ at the nanoscale, magnetic anisotropy normally increases as particle size decreases. The measured magnetic characteristics in this study, on the other hand, revealed that anisotropy rose with particle size, implying that the magnetocrystalline component played a substantial role, as seen in Fe_3_O_4_ and CFO NPs [77]. 

**Figure 10:**
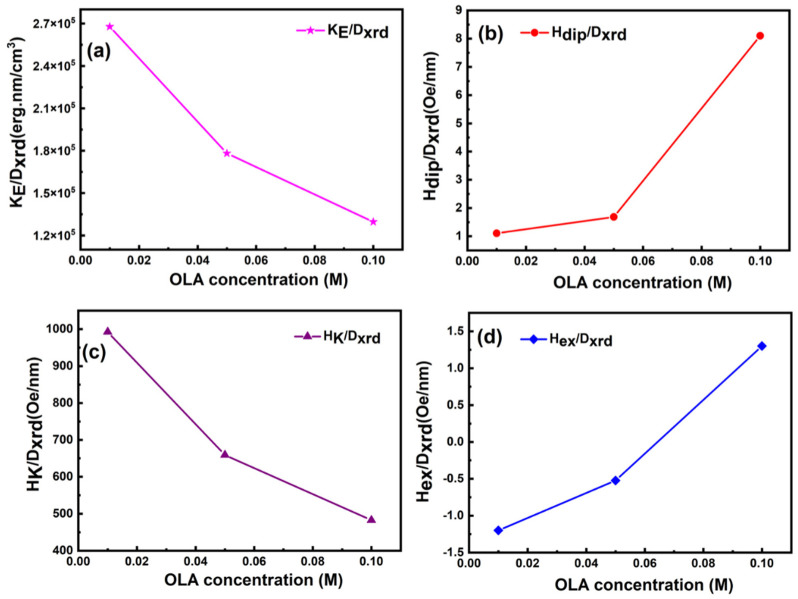
KE/Dxrd (**a**), Hdip/Dxrd (**b**), HK/Dxrd (**c**) and Hex/Dxrd (**d**) versus OLA concentration of functionalized CoFe2O4 NPs at 10 K.

The functionalization of CFO NPs with OLA alters the interparticle magnetic interaction; therefore, the strength of dipolar interparticle interactions in the samples was estimated by the maximal dipolar field H*_dip_* between nearest-neighbor particles [27,77]. H*_dip_* appeared to increase with an increase in OLA concentration (Figure 10b). Because of the higher magnetic particle size at different concentrations of OLA, H*_dip_* (Table 3) rapidly increased at 300 K compared to 10 K.

Besides, the shift of the M-H loop generally referred to as the exchange bias field (Hex), has been seen in ferromagnetic NPs [27]. The M-H loop shifts along the field axis at 10 K, and this shift increases with OLA concentration. Because of the rise in surface/volume ratio (S/V), interface exchange coupling increased as particle size decreased (Figure 10d). The OLA coating [74] lowered spin disorder, making interface exchange coupling between the ordered core and the magnetically disordered shell more challenging. Furthermore, the rise of interparticle interactions, which improved the efficiency of the exchange bias phenomena, can be attributed to an increase in the exchange bias field for sample CoFe3 [77].

## 4. Conclusions

The CoFe_2_O_4_ nanoparticles in a size range of 14–20 nm were synthesized by varying oleylamine concentration. The structure-property analyses indicated that the effect of oleylamine concentration was significant on the structure, morphology, inter-particle interactions, electronic structure, and magnetic properties. All the CFO NPs crystallized in the cubic spinel structure with a lattice constant lower than that of bulk CFO. The interaction of oleylamine with CoFe_2_O_4_ surface atoms altered magnetic characteristics such as maximum saturation magnetization, remanent magnetization, coercivity, effective anisotropy constant, and interparticle interactions significantly. Maximum saturation magnetization (M_S_ = 82.84 emu/g) and remanent magnetization (M_r_ = 28.01 emu/g) values were obtained for the M_S_ and M_r_ values due to OLA-induced NP-size variation. Similarly, at 10 K, all the OLA functionalized CFO NPs exhibited remarkably higher coercivity (H_C_) of 6.09–8.00 kOe compared to bulk CFO. Understanding the effect of oleylamine in regulating the nucleation and the structure-morphology-magnetic property, correlations established provide a roadmap to produce CFO NPs with desired size and properties for a given application.

## Figures and Tables

**Table 1:** The number of moles of all reagents used in the synthesis of CFO NPs.

Reagent	Moles
cobalt (II) nitrate	0.001
iron (III) nitrate	0.002
Urea	0.002
ethylene glycol	2.5
Oleylamine	0.01 M (0.46 mL), 0.05 M (2.3 mL), 0.1 M (4.6 mL)

**Table 2:** Bond angle and bond length extracted from Rietveld refinement of the samples.

Bond Angle (°)	CoFe1	CoFe2	CoFe3	Bond Length (Å)	CoFe1	CoFe2	CoFe3
O–Co2–O	109.47	109.47	109.47	*d*_12_ = O–Co2	1.855	1.953	1.881
				*d*_23_ = Co2–O	1.855	1.953	1.881
				*d*_13_ = O–O	3.029	3.189	3.072
Co1–O–Co2	124.33	122.01	123.80	*d*_12_ = Co1–O	2.071	2.015	2.061
				*d*_23_ = O–Co2	1.855	1.953	1.881
				*d*_13_ = Co1–Co2	3.473	3.471	3.478
O–Co1–O	88.67	85.31	87.91	*d*_12_ = O–Co1	2.071	2.015	2.061
				*d*_23_ = Co1–O	2.071	2.015	2.061
				*d*_13_ = O–O	2.894	2.731	2.861
O–Co1–O	91.33	94.69	92.09	*d*_12_ = O–Co1	2.071	2.015	2.061
				*d*_23_ = Co1–O	2.071	2.015	2.061
				*d*_13_ = O–O	2.962	2.964	2.967

**Table 3:** Magnetic parameters obtained for CoFe1, CoFe2, CoFe3 and CoFe3-_350_ samples.

Sample	Temperature (K)	M_S_ (emu/g)	M_r_ (emu/g)	M_r_/M_S_	H_C_ (Oe)	H_ex_ (Oe)	K_E_ (erg/cm^3^)	H_dip_ (Oe)
CoFe1	10	101.79	80.65	0.79	6090.83	−23.09	5.14 × 10^6^	21.24
	300	82.84	28.01	0.34	421.35	−31.59	3.41 × 10^6^	377.29
CoFe2	10	51.31	36.89	0.72	8007.88	−10.62	1.93 × 10^6^	32.25
	300	42.295	16.78	0.39	658.81	29.24	2.89 × 10^5^	475.59
CoFe3	10	35.57	26.615	0.75	6575.89	19.35	2.31 × 10^5^	120.57
	300	30.23	9.615	0.32	459.87	−10.31	1.15 × 10^5^	2028.75
CoFe3-_350_	10	80.06	66.72	0.83	12,465.6	−36.00	8.23 × 10^6^	13,051.54
	300	73.24	31.89	0.44	1210.03	−05.13	7.31 × 10^5^	9140.98

**Table 4:** Comparison of the saturation magnetization and coercivity of OLA produced NPs. with other ferrites in the literature.

Composition	Magnetization (M_S_; emu/g)	Coercivity (H_C_; Oe)	Reference(s)
CoFe_2_O_4_	54.65	8.19	[4]
Co_0.5_Mn_0.5_Fe_2_O_4_	55.32	9.05	[4]
NiFe_2_O_4_	19	-	[5]
Co_0.5_ Zn_0.5_ Fe_2_O_4_	52.03	82.71	[6]
CuFe_2_O_4_	20.62	63.1	[7]
ZnFe_2_O_4_	24.05	-	[8]
MnFe_2_O_4_	51.99	-	[9]
MnFe_2_O_4_	46	64	[10]
Mg_1_Fe_2_O_4_	0.071	194	[11]
Zn*_0.5_*Mg_0.5_Fe_2_O_4_	0.293	69	[11]

## Data Availability

The data that support the findings of this study are available from the corresponding author upon request.

## Supplementary Materials

The following supporting information can be downloaded at: https://www.mdpi.com/article/10.3390/nano12173015/s1, Figure S1: Energy dispersive X-ray spectroscopy (EDS) patterns for CoFe1 (a), CoFe2 (b) and CoFe3 (c) samples; Figure S2: Histogram for CoFe1 (a), and CoFe2 (b) samples along with log-normal function showing the size distribution of nanoparticles; Figure S3: FTIR spectra of pure oleylamine; Figure S4: XPS survey spectra of CFO NPs; Table S1: Structural parameters obtained from SAXS; Table S2: Infrared vibrational assignments for the pure oleylamine; Table S3: Structural parameters obtained from Rietveld refinement of XRD; Table S4: Atomic position, occupancy, and agreement factors obtained for CoFe1, CoFe2, and CoFe3 from Rietveld Refinement.
