# Supplementary material for: Effect of Oleylamine on the Surface Chemistry, Morphology, Electronic Structure, and Magnetic Properties of Cobalt Ferrite Nanoparticles
Source: Nanomaterials (Basel). 2022 Aug 31;12(17):3015. doi: 10.3390/nano12173015 (PMC9458106; doi:10.3390/nano12173015)
Supplement: Supplementary file 1 [file nanomaterials-12-03015-s001.zip › nanomaterials-1763572-supplementary.pdf]

nanomaterials

Article

Effect of Oleylamine on the Surface Chemistry, Morphology, Electronic Structure, and Magnetic Properties of Cobalt Ferrite Nanoparticles

Sumayya M. Ansari [1], Bhavesh B. Sinha [2], Debasis Sen [3,4], Pulya U. Sastry [3,4], Yesh D. Kolekar [1,*] and C. V. Ramana [5,*]

[1] Department of Physics, Savitribai Phule Pune University, Pune 411 007, Maharashtra, India
[2] National Center for Nanoscience and Nanotechnology, University of Mumbai, Mumbai 400 032, Maharashtra, India
[3] Bhabha Atomic Research Centre (BARC), Solid State Physics Division, Mumbai 400 085, Maharashtra, India
[4] Homi Bhabha National Institute, Anushaktinagar, Mumbai 400 094, Maharashtra, India
[5] Centre for Advanced Materials Research (CMR), University of Texas, El Paso, TX 79968, USA
* Correspondence: ydkolekar@gmail.com (Y.D.K.); rvchintalapalle@utep.edu (C.V.R.)

1. EDS

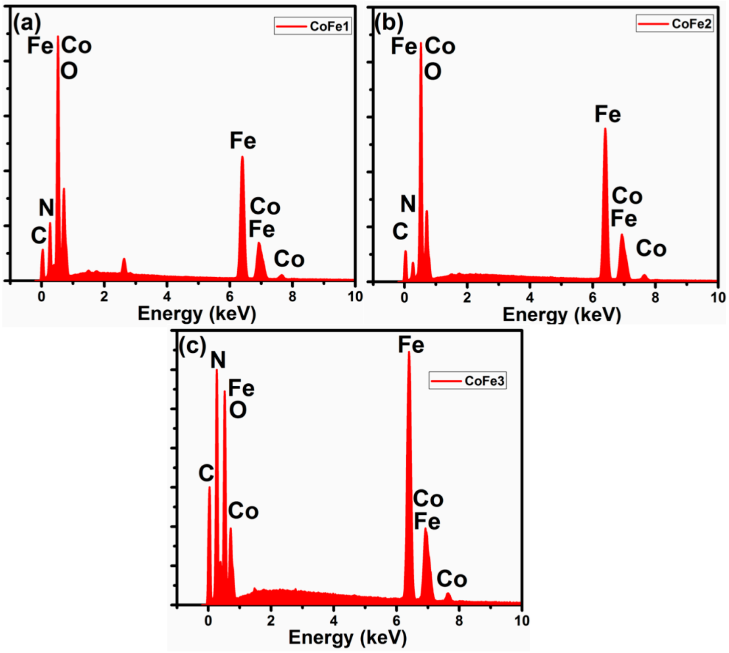

Figure S1. Energy dispersive X-ray spectroscopy (EDS) patterns for CoFe1 (**a**), CoFe2 (**b**) and CoFe3 (**c**) samples.

2. SEM

The particle size distribution is calculated from a FESEM micrograph by plotting the histogram and fitted with a log-normal function.

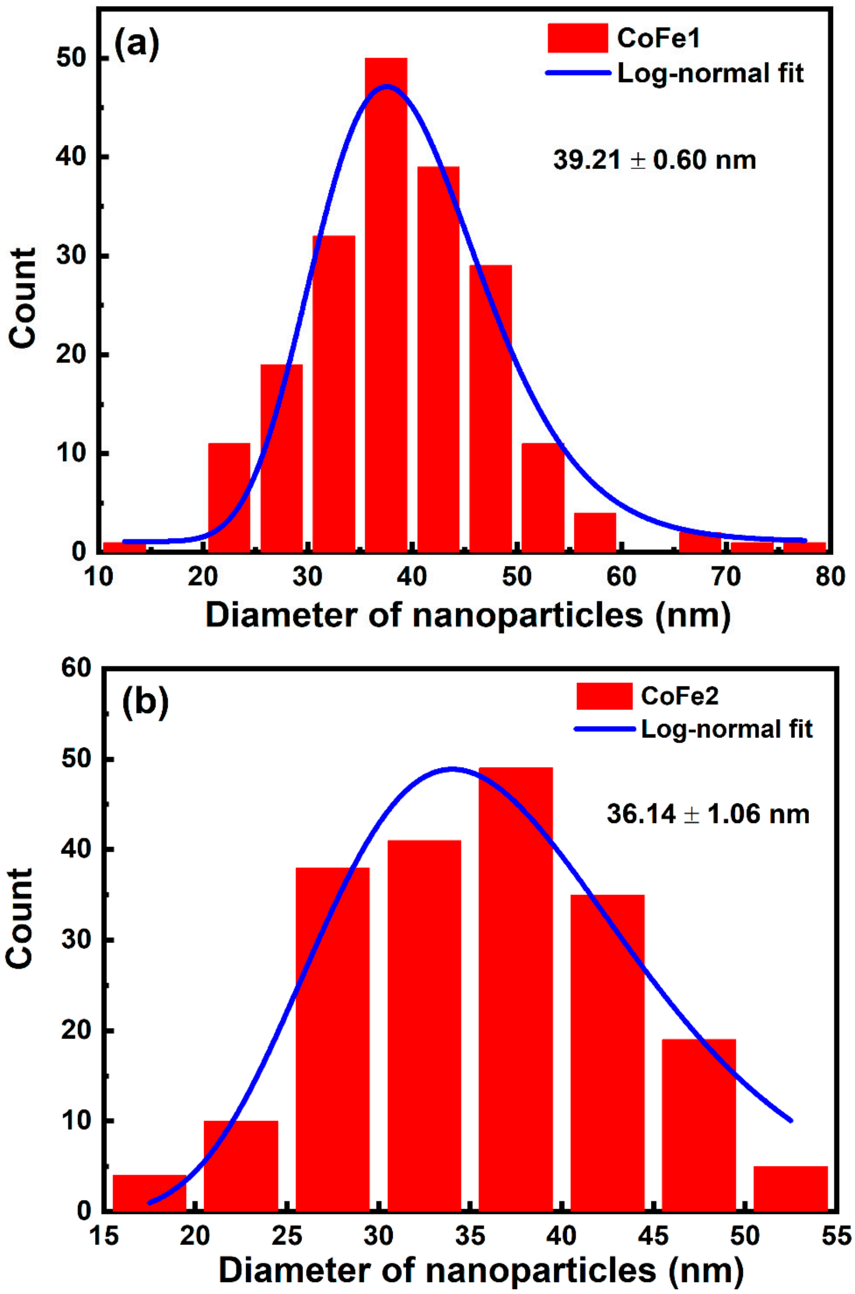

Figure S2. Histogram for CoFe1 (**a**), and CoFe2 (**b**) samples along with log-normal function showing the size distribution of nanoparticles.

3. SAXS Analysis

Small-angle X-ray scattering (SAXS) measurements were carried out using a Rigaku small-angle goniometer mounted on a rotating anode X-ray generator. Scattered X-ray intensity I (q) was recorded using a scintillation counter with a pulse height analyzer by varying the scattering angle 2q where *q* is the scattering vector given by 4p.sin (q) / λ and λ is the wavelength of incident (Cu Kα) X-rays. The intensities were corrected for sample absorption and smearing effects of collimating slits [1].

Table S1. Structural parameters obtained from SAXS.

Parameter \ Samples	CoFe1	CoFe2	CoFe3
Outer radius (R_1, nm)	5.48	4.00	3.05
Inner radius (R_2, nm)	3.5	1.5	1.0
The polydispersity index (σ, nm)	0.36	0.31	0.29

The radius of the monomer (r_o, nm)	5.61	3.49	2.17
Size of aggregate (x_i, nm)	11.77	13.52	27.69
Fractal dimension (D)	3.00	3.00	3.00

4. FTIR

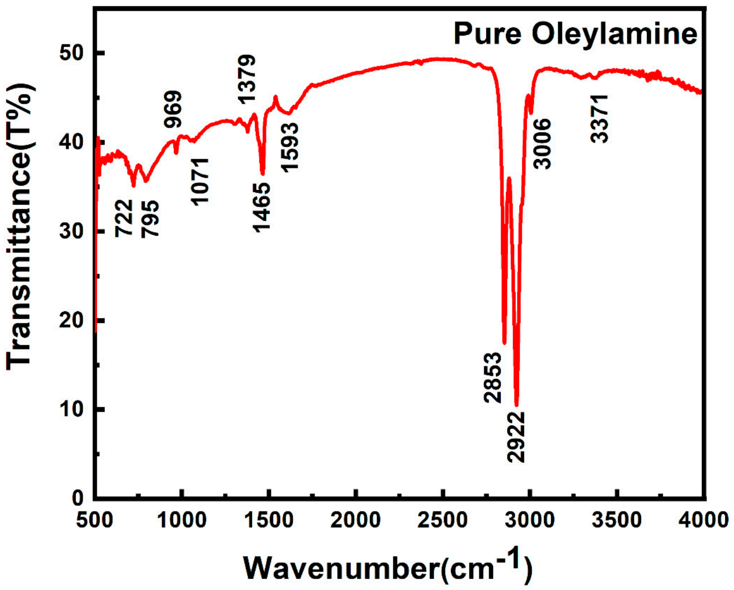

Figure S3. FTIR spectra of pure oleylamine.

Table S2. Infrared vibrational assignments for the pure oleylamine.

Vibrational modes	Frequency (cm^{-1})
ν_{as} (NH_2)and ν_s (NH_2)	3376, 3300
δ(=C−H)	3006
ν_{as} (C−H)and ν_s (C−H)	2922, 2854
δ(−C=C)	1647
δ (NH_2)	1593, 795
δ (CH_3)	1465
δ (C−N)	1071
δ (C−C)	722
ν_{as}– asymmetric stretching vibration; ν_s– symmetric stretching vibration; δ– Bending vibration	

5. XRD

Table S3. Structural parameters obtained from Rietveld refinement of XRD.

Sample	Average crystallite size (nm)	Lattice parameter (*a*, Å)	X-ray density ($d_{X\text{-}ray}$) (gm/cm^3)
CoFe1	19.18	8.3769	5.301
CoFe2	19.14	8.3719	5.311
CoFe3	14.88	8.3894	5.278

Table S4. Atomic position, occupancy, and agreement factors obtained for CoFe1, CoFe2, and CoFe3 from Rietveld Refinement.

Atom	Atomic position			Occupancy	Agreement factors
	x	y	z		
Sample CoFe1					

Co1	0.000	0.000	0.000	0.0300	R_{wp}= 52.10
Fe1	0.000	0.000	0.000	0.0533	R_{exp}= 50.97
Co2	0.625	0.625	0.625	0.0133	R_B= 1.186
Fe2	0.625	0.625	0.625	0.0283	S = 1.0221
O1	0.247	0.247	0.247	0.1785	χ^2= 1.04
		Sample CoFe2			
Co1	0.000	0.000	0.000	0.0300	R_{wp}= 63.70
Fe1	0.000	0.000	0.000	0.0533	R_{exp}= 63.26
Co2	0.625	0.625	0.625	0.0133	R_B= 1.142
Fe2	0.625	0.625	0.625	0.0283	S = 1.006
O1	0.240	0.240	0.240	0.1983	χ^2= 1.01
		Sample CoFe3			
Co1	0.000	0.000	0.000	0.033	R_{wp}= 16.70
Fe1	0.000	0.000	0.000	0.053	R_{exp}= 15.98
Co2	0.625	0.625	0.625	0.013	R_B= 1.243
Fe2	0.625	0.625	0.625	0.028	S = 1.045
O1	0.245	0.245	0.245	0.1769	χ^2= 1.10

R_{wp}: weighted profile factor, R_{exp}: expected weighted profile factor, R_B: Bragg factor,.
S: Goodness of fit = R_{wp}/R_{exp}, reduced chi-square: (χ^2).

6. XPS

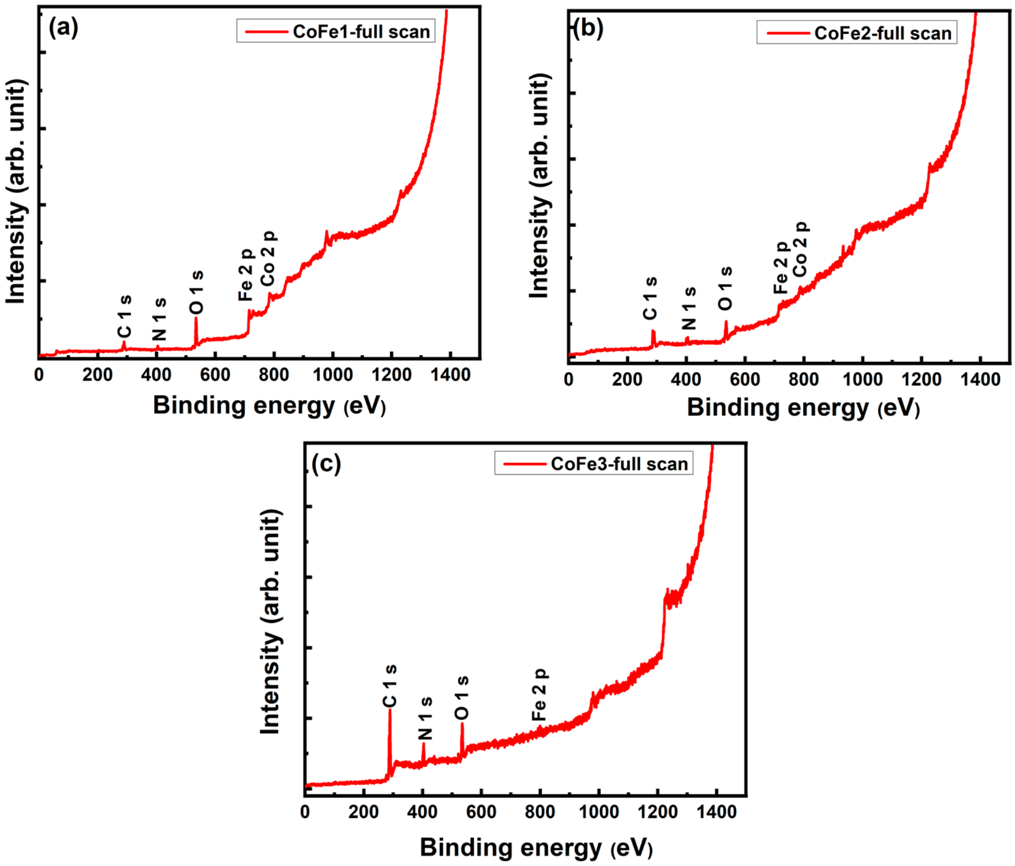

Figure S4. XPS survey spectra of CFO NPs.

7. Thermal Behavior - Thermogravimetric (TGA) Analysis

The number of OLA molecules per particle can be determined using the following formula [2] based on the weight loss.

$$N = \frac{W\ N_0\ \rho\,(4/3)\pi\ R^3 \times 10^{-23}}{M} \tag{1}$$

Where N is the number of surfactant molecules (ligands) per particle, W is the weight loss in percent (%), N_0 is Avogadro's number, ρ is the density of NPs, R is the average radius of the CFO NPs (obtained from SAXS analysis), and M is the molecular weight of OLA (267.49 g/mol).

References

1. Ahrenstorf, K.; Heller, H.; Kornowski, A.; Broekaert, J.A.C.; Weller, H. Nucleation and Growth Mechanism of NixPt1−x Nanoparticles. *Adv. Funct. Mater.* **2008**, *18*, 3850–3856, doi:https://doi.org/10.1002/adfm.200800642.
2. Ansari, S.M.; Sinha, B.B.; Phase, D.; Sen, D.; Sastry, P.U.; Kolekar, Y.D.; Ramana, C.V. Particle Size, Morphology, and Chemical Composition Controlled CoFe2O4 Nanoparticles with Tunable Magnetic Properties via Oleic Acid Based Solvothermal Synthesis for Application in Electronic Devices. *ACS Appl. Nano Mater.* **2019**, *2*, 1828–1843, doi:10.1021/acsanm.8b02009.
